# A Protein Intrinsic Disorder Approach for Characterising Differentially Expressed Genes in Transcriptome Data: Analysis of Cell-Adhesion Regulated Gene Expression in Lymphoma Cells

**DOI:** 10.3390/ijms19103101

**Published:** 2018-10-10

**Authors:** Gustav Arvidsson, Anthony P. H. Wright

**Affiliations:** Clinical Research Center, Department of Laboratory Medicine, Karolinska Institutet, Huddinge SE 141 57, Sweden; gustav.arvidsson@ki.se

**Keywords:** intrinsic disorder, intrinsic disorder prediction, intrinsically disordered region, protein conformation, transcriptome, RNA sequencing, Microarray, differentially regulated genes, gene ontology analysis, functional analysis

## Abstract

Conformational protein properties are coupled to protein functionality and could provide a useful parameter for functional annotation of differentially expressed genes in transcriptome studies. The aim was to determine whether predicted intrinsic protein disorder was differentially associated with proteins encoded by genes that are differentially regulated in lymphoma cells upon interaction with stromal cells, an interaction that occurs in microenvironments, such as lymph nodes that are protective for lymphoma cells during chemotherapy. Intrinsic disorder protein properties were extracted from the Database of Disordered Protein Prediction (D^2^P^2^), which contains data from nine intrinsic disorder predictors. Proteins encoded by differentially regulated cell-adhesion regulated genes were enriched in intrinsically disordered regions (IDRs) compared to other genes both with regard to IDR number and length. The enrichment was further ascribed to down-regulated genes. Consistently, a higher proportion of proteins encoded by down-regulated genes contained at least one IDR or were completely disordered. We conclude that down-regulated genes in stromal cell-adherent lymphoma cells encode proteins that are characterized by elevated levels of intrinsically disordered conformation, indicating the importance of down-regulating functional mechanisms associated with intrinsically disordered proteins in these cells. Further, the approach provides a generally applicable and complementary alternative to classification of differentially regulated genes using gene ontology or pathway enrichment analysis.

## 1. Introduction

Genome-wide approaches to identify genes that are differentially expressed under different conditions of interest have become a standard approach to investigating mechanisms involved in biological processes. The analysis pipeline used in such studies generally leads quickly to some form of gene ontology analysis, in order to identify biological functions that are associated with the differentially regulated genes. A complementary approach would be to analyse differentially regulated genes in relation to predicted conformational properties of the proteins they encode but such an approach has not been reported.

Characterisation of differentially regulated genes in relation to predicted or known conformational properties of the proteins they encode would be of interest in the light of recent discoveries showing overall relationships between conformational properties and different types of protein functionality or mechanism of action [1]. For example, the catalytic domains of enzymes are generally ordered globular conformations while transcription factors are characterised by a preponderance of intrinsic disorder leading to ensembles of many alternative conformational forms [2]. It is now clear that about half the proteins in eukaryotes contain at least one extended (>30 amino acid residues) intrinsically disordered region (IDR) and some proteins are completely disordered [3].

Interestingly, IDRs occur more frequently in regulatory proteins and disease-related proteins [4,5]. We recently identified genes that are differentially expressed in mantle cell lymphoma (MCL) cells that adhere to stromal cells with which they are co-cultured compared to non-adherent MCL cells in the same culture [6]. The differentially regulated gene set defined in this in vitro model system showed substantial overlap with genes that are differentially regulated in the lymph node microenvironment of MCL and chronic lymphoblastic leukaemia (CLL) patients. Retention of lymphoma cells in microenvironments is thought to lead to minimal residual disease, in which a subpopulation of cancer cells receives survival signals from normal cells in microenvironments, thus allowing them to survive during treatment and to subsequently cause disease relapse. In vitro, minimal residual disease is mimicked by cell adhesion mediated drug resistance whereby, for example, lymphoma cells residing in close proximity to stromal cells manifest an enhanced level of resistance to cytostatic drugs [7]. Thus, differentially regulated genes in co-cultured adherent lymphoma cells are likely to represent processes important for cell adhesion mediated drug resistance and minimal residual disease.

In our recent study, we identified 1050 genes that were differentially regulated in MCL cells adhered to stromal cells compared to non-adherent MCL cells in the same co-culture. The four main functional themes characterised by the differentially regulated gene set were cell adhesion, anti-apoptosis and B-cell signalling/immune-modulation, associated with up-regulated genes in adherent cells, as well as early mitotic processes, associated with down-regulated genes [6]. Here we test whether the differentially regulated gene set or its subsets encode proteins that differ in IDR properties compared to non-regulated genes.

## 2. Results

**Figure 1:**
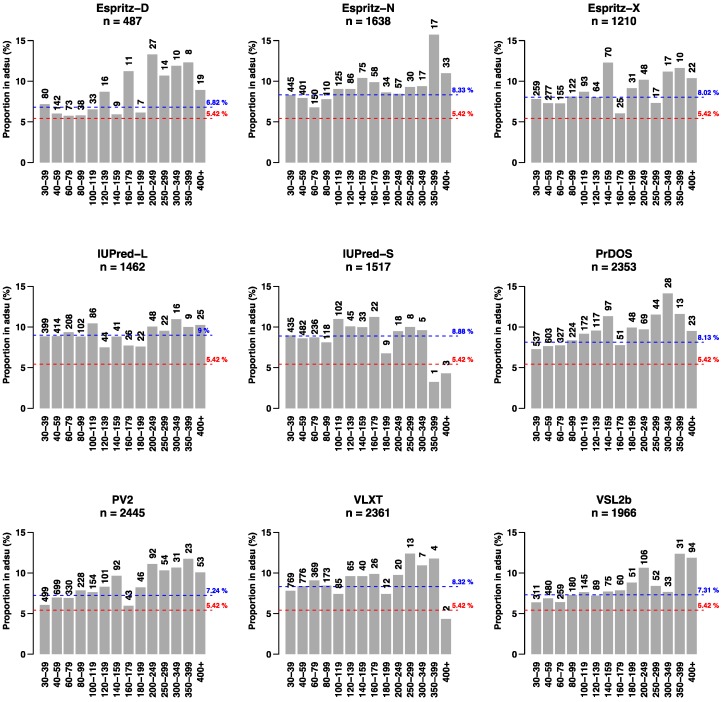
Enrichment of intrinsically disordered regions (IDRs) in proteins encoded by genes that are differentially expressed in lymphoma cells upon adhering to stromal cells. The number (*n*) of IDRs (≥30 residues) for each predictor is shown as well as how the detected IDRs are distributed in relation to length. The number of IDRs in each size category is shown. The blue line shows the percentage of all IDRs encoded by adhesion-related genes (adsu) and non-adhesion-related genes (nadsu) that are associated with the adsu set, while the red line shows the percentage expected if IDRs are equally distributed between the adsu and nadsu sets.

To determine whether there might be a difference in the frequency of IDRs (defined as predicted IDRs ≥ 30 amino acid residues in length) in proteins encoded by adhesion-regulated genes (adsu, *n* = 1009) compared to other proteins (nadsu, *n* = 17,612), we calculated the percentage of IDRs in adhesion-related proteins for each IDR predictor (Figure 1, blue line) and compared it to the proportion of genes in the adhesion-regulated gene set (5.4%, Figure 1, red line). For all predictors, the proportion of predicted IDRs associated with the adhesion gene set exceeded the frequency expected based on the proportion of proteins in the set. For many predictors, Figure 1 also shows a tendency towards a larger number of longer IDRs in proteins encoded by the adhesion gene set at the expense of shorter IDRs.

To determine whether the enhanced frequency of IDRs in proteins encoded by adhesion-regulated genes was significant, we used a resampling approach to test whether the IDR frequency associated with the 1009 adhesion-regulated genes lay outside the distribution of frequencies generated by 1000-fold resampling of 1009 genes from the control gene set (*n* = 17,612). A *z*-score and associated *p*-value was generated for data from each predictor. As shown in Table 1 (adsu vs. nadsu), the enrichment of IDRs in proteins encoded by adhesion-regulated genes (adsu) was significant for all predictors.

Next, we tested whether the enrichment of IDRs associated with the adhesion-regulated gene set could be ascribed to subsets of the adhesion-regulated genes. Comparison of proteins encoded by genes manifesting a greater degree of regulation (fold change ≥ 1.3) relative to the remaining regulated genes showed fewer IDRs in more highly regulated genes compared to less highly regulated genes for all predictors and with lower levels of significance compared to the comparison of regulated and non-regulated genes (data not shown). Thus, there is an enrichment of IDRs in adhesion-regulated genes but the enrichment is not related to the extent of their regulation. Comparison of the up-regulated subset (adsu_up, change >1) relative to the down-regulated subset (adsu_down, change <1), on the other hand, showed an enhanced enrichment of IDRs in proteins encoded by the adsu_down subset compared to the enhancement levels in Figure 1, with high levels of significance (Table 1, adsu_down vs. adsu_up). Thus, the enrichment in IDRs in proteins encoded by adhesion-regulated genes is mainly associated with proteins encoded by down-regulated genes.

We next investigated whether the length of IDRs in proteins encoded by adsu genes tends to be longer than in other proteins (nadsu). As expected, IDR length is not normally distributed, as indicated by the consistently higher value of the mean compared to the median (Table 2), as well as tests of normality (data not shown). Thus, a Mann–Whitney test was used to test the significance of differences in IDR length between groups. Table 2 shows that some predictors (notably PV2, PrDOS and VSL2b) predict longer IDRs in proteins encoded by adsu genes that in other proteins (nadsu), but for other predictors the difference is less significant or lacking in statistical support. IDRs encoded by adsu_down genes were significantly longer than IDRs encoded by adsu_up genes for all predictors. Thus, IDRs in proteins encoded by genes that are down-regulated in adherent cells tend to be both more frequent and longer than IDRs in other proteins.

We next addressed how IDRs are distributed among the proteins encoded by the adsu_down gene set in relation to proteins associated with the adsu_up and nadsu gene sets (Table 3).

**Figure 2:**
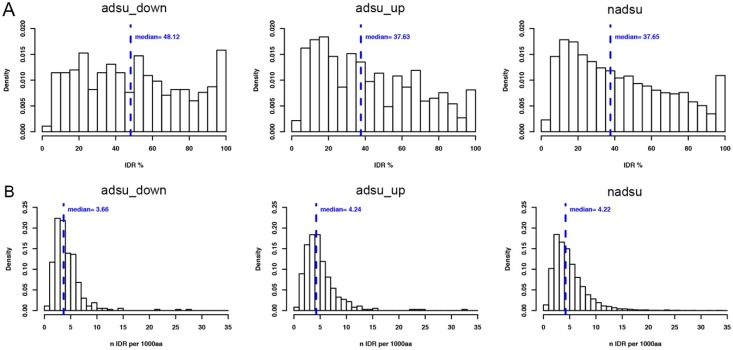
Relative frequency distributions of proportion of IDR per protein and length-normalized number of IDRs per protein for proteins encoded by adsu_down genes in relation to adsu_up and nadsu genes. IDR predictions were made using VSL2b. (**A**) Relative frequency distributions (Density) of IDR-containing proteins with different percent IDR content. The median position and value are shown in blue. (**B**) Relative frequency distributions (Density) of numbers of IDRs per IDR-containing protein, normalized for differences in protein length (IDR number per 1000 amino acid residues). The median position and value are shown in blue.

The proportion of completely disordered proteins was higher for the adsu_down sets than for proteins encoded by the other gene sets, as was the proportion of proteins containing at least one IDR. The median proportion of the protein sequences that were predicted as IDR was higher for the adsu_down group, irrespective of whether all proteins were considered or only proteins containing IDRs. Table 3 shows data for the VSL2b predictor but other predictors generally produced a similar result, especially PrDOS and PV2. The frequency of IDR-containing proteins with different IDR proportions for the different gene sets is compared graphically in Figure 2A.

For proteins encoded by nadsu and adsu_up, the relative frequency declines progressively as the proportion of IDR per protein increases. Contrastingly, a more even distribution of relative frequencies is seen for adsu_down proteins, with relatively fewer low-IDR content proteins and an increased proportion of high-IDR content proteins. Interestingly, the protein length-normalized number of IDRs per protein is somewhat lower for proteins encoded by adsu_down genes, compared to adsu_up and nadsu genes (Figure 2B). Thus, the greater IDR content of adsu_down encoded genes tends to be associated with fewer and longer IDRs when only IDR-containing proteins are analyzed.

**Figure 3:**
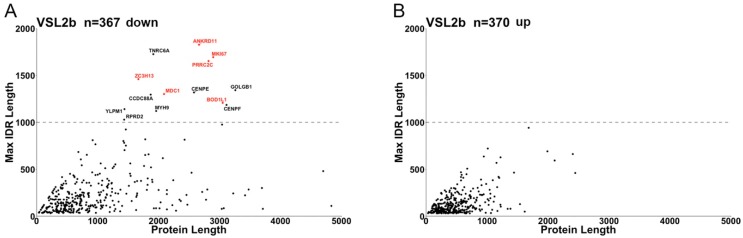
Comparison of proteins encoded by down- or up-regulated adhesion-regulated genes with regard to longest IDR length per protein and protein length. IDR-containing proteins encoded by (**A**) down-regulated adhesion-regulated genes (adsu_down) and (**B**) up-regulated adhesion-regulated genes (adsu_up) are shown. Of the 14 proteins in (**A**) for which the maximum IDR length is greater than 1000 residues (above dotted line), 6 proteins (red text) were also found in the sets of 14 proteins with the longest IDRs predicted by the PV2 and PrDOS predictors.

**Figure 4:**
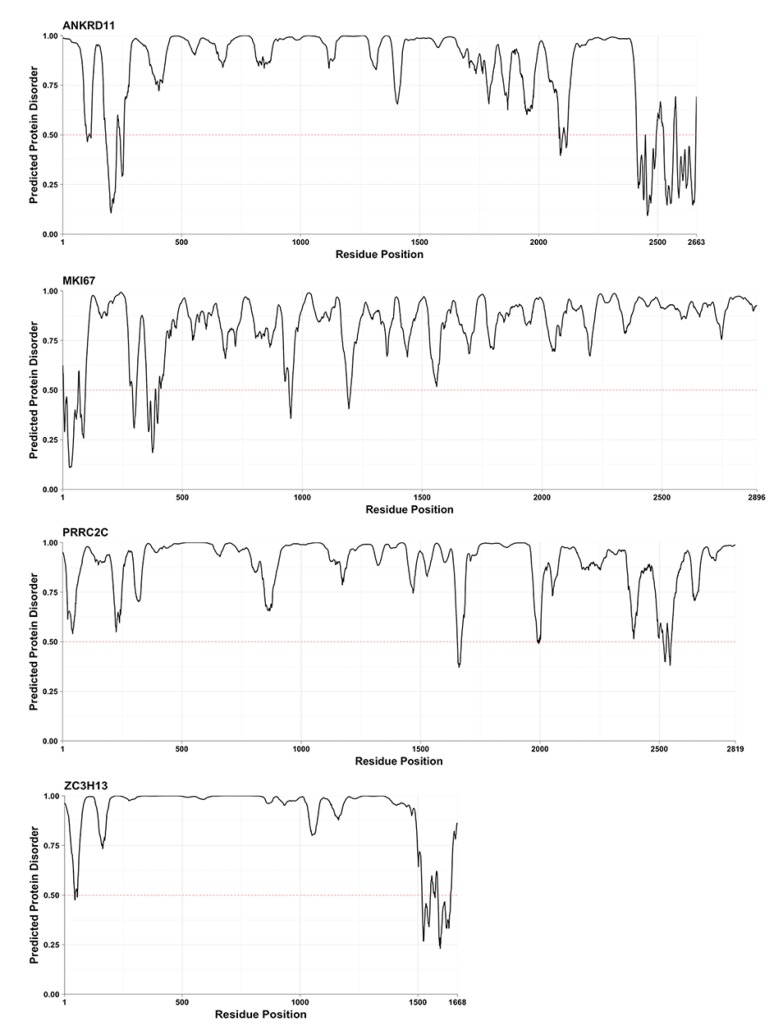

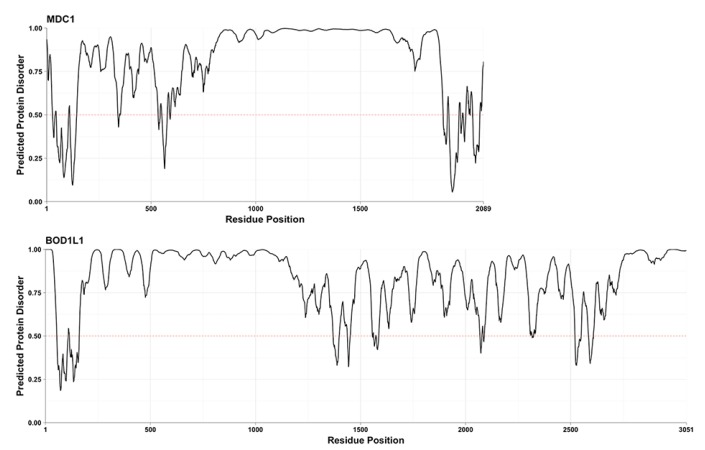
Examples of proteins with long IDRs. Proteins that are reproducibly found by the VSL2b, PV2 and PrDOS predictors in the set of 14 proteins with the longest predicted IDRs (red text in Figure 3A) are shown. The residue-by-residue intrinsic disorder score (VSL2b) is plotted as a function of residue number throughout the length of the respective proteins. The horizontal gridline at a score of 0.5 distinguishes regions predicted to be ordered (<0.5) or intrinsically disordered (>0.5).

To further investigate differences in IDR lengths between groups, we plotted the length of the longest IDR in each protein as a function of protein length to compare adsu_down and adsu_up encoded proteins (Figure 3).

adsu_down encoded proteins are characterized by both longer protein length and longer length of the longest IDR (VSL2b). There are 14 adsu_down encoded proteins with IDRs longer than 1000 residues and these are also among proteins with the longest IDRs for most other predictors (notably PrDOS and PV2). The IDR score profiles for the 6 proteins that are reproducibly found in the top 14 proteins with longest IDRs by the VSL2b, PV2 and PrDOS predictors (red text in Figure 3A) are shown in Figure 4.

Consistent with Figure 3A, most of the proteins are predicted to be disordered throughout most of their length. Some contain extended regions with close to maximal intrinsic disorder scores (e.g., ZC3H13), while others are characterized by fluctuating levels of intrinsic disorder (e.g., MKI67). Some proteins contain both patterns in different regions of the protein (e.g., BOD1L1). Many of the proteins have short regions that are predicted to be ordered and that could correspond to folded protein domains. The different types of predicted conformation could inform about mechanisms involved in the function of proteins encoded by down-regulated genes in relation to up-regulated genes (see Discussion).

## 3. Discussion

The main finding of this work is that proteins encoded by genes that are down-regulated in lymphoma cells upon adhering to stromal cells, typically found in microenvironments that increase cancer-cell survival, tend to have more frequent and longer regions of predicted intrinsically disordered conformation than proteins encoded by up-regulated genes or other expressed genes in the same cells. Our previous work has shown that many proteins encoded by down-regulated genes in adherent cells are involved in early stages of mitosis [6]. The present results complement this observation by suggesting that proteins encoded by the down-regulated gene set tend to function by mechanisms that are associated with intrinsically disordered regions. A secondary finding is that many of the proteins encoded by down-regulated genes are larger than proteins encoded by up-regulated genes.

Intrinsically disordered protein regions can be broadly divided into regions that are always disordered and disordered regions that form one or more ordered conformations in particular molecular environments, such as during coupled binding and folding interactions with partner proteins [8]. Some IDRs have been shown to bind partners in the disordered state via multi-valent interactions, mediated by short linear motifs that are distributed along the length of the IDR [5,9,10,11]. However, IDRs have other functions in addition to interaction with partners. One such function is mediation of phase transitions in cells that allow for compartmentalization of cellular regions in so-called “membrane-less organelles” that include nucleoli, nuclear speckles, P-bodies and chromatin [12,13,14,15,16]. These kinds of functional mechanisms might be associated with the IDRs that have consistently close-to-maximal prediction scores over extended regions of proteins encoded by down-regulated genes, as exemplified by some of the proteins in Figure 3 and Figure 4.

The clearest example of a protein that is predicted to be maximally disordered throughout most of the protein sequence is ZC3H13. Interestingly, ZC3H13 is part of the WTAP complex, which is involved in RNA splicing and processing and is localized in nuclear speckles [17]. It is likely that such speckles result from phase transition processes and it is possible that the disordered region of ZC3H13 is important for speckle formation or ZC3H13 localization to the speckle. In fact, many documented types of so-called proteinaceous membrane-less organelles are located in the nucleus and include chromatin in addition to nuclear speckles, nucleoli and many other bodies [14]. The MDC1 protein (Figure 3 and Figure 4) contains a central region predicted to be completely disordered, flanked by less disordered/structured regions, which are known to mediate binding to several partner proteins at chromatin regions containing double-stranded DNA breaks [18]. Thus, MDC1 has been regarded as a “scaffold” protein responsible for spreading of DNA-repair factors over the damaged chromatin region and it is tempting to speculate that the central disordered region could play a role in phase-transitions. Other proteins in Figure 3 and Figure 4 that have extensive regions predicted to be completely disordered and that work in a chromatin environment are YLPM1, involved in regulating telomerase activity, and BOD1L1, a protein that protects stalled DNA replication forks.

MKI67 is predicted to be disordered (with varying score) throughout almost its entire length (see Figure 4). Interestingly, MKI67 orchestrates formation of the perichromosomal layer, which coats the condensed chromosomes during mitosis in order to prevent chromosome aggregation [19]. In mitotic mammalian cells, the nuclear membrane and nucleolus are broken down and nucleolar proteins including the known phase-transition proteins, Nucleophosmin and Fibrillarin, that drive nucleolus formation in interphase cells [20], are also found in the mitotic perichromosomal layer. This fact, taken together with the RNA-binding activity associated with MKI67, suggests that the perichromosomal layer may be formed by phase transition phenomena. Interestingly, higher expression of MKI67 is a negative prognostic marker for MCL patients [21].

In the IDR class that conditionally adopts ordered conformations in some molecular contexts, the ordered conformations are characterized by varying degrees of “fuzziness”, defined as the existence of a heterogeneous range of ordered conformations in the context of, for example, interaction with a single partner [22]. Many proteins that conditionally adopt ordered conformations contain pre-structure motifs (PreSMos), defined as short protein regions within IDRs that have a weak propensity for secondary structure formation leading to formation of unstable secondary structure elements in a minority sub-population of IDR-containing proteins [23]. PreSMos become stabilized during coupled binding and folding, and form part of the folded protein conformation that is seen in complexes with partner proteins. Protein regions encoded by down-regulated genes that show alternating sub-regions of higher and lower intrinsic disorder scores might correspond to these kinds of IDR since the short regions with lower intrinsic disorder scores may represent PreSMos. The CENPE and CENPF proteins are characterized by disordered regions interspersed with regions with lower disorder scores that could represent regions containing PreSMos. This would be consistent with the multiple interactions made by these proteins within the kinetochore structure that binds to the centromeric chromatin of chromosomes during mitosis. TNRC6A is a member of the GW182 family of scaffold proteins that are important for organization of proteins needed for RNA-mediated gene silencing and are found in P-bodies that are formed by a phase transition process [24].

Although somewhat speculative, the preceding sections suggest mechanisms by which some of the large proteins with large amounts of intrinsic disorder might contribute the propagation of lymphoma cells in suspension as well as how their down-regulation could lead to reduced proliferation of lymphoma cells adhered to stromal cells. Reduced proliferation is known to increase the survival of cancer cells during chemotherapy, which primarily targets proliferating cells [7,25]. Further, the cell cycle arrest that occurs in adherent MCL cells [26] would be expected to reduce the need for apoptotic responses and we previously showed that adherence to stromal cells is associated with up-regulation of anti-apoptotic genes [6].

We have shown that predicted intrinsic disorder can be used to interrogate proteins encoded by transcriptome data and that identification of gene sets encoding proteins with characteristic predicted disorder properties can provide information relevant for understanding the mechanisms underlying the functionality of groups of proteins. This approach complements the commonly used gene ontology analysis approach, which primarily gives information about the cellular components or processes that are characteristic for the function of protein sets. Both approaches provide information that can be used for hypothesis building and the design of further experiments.

In this work, we have only analyzed predicted protein disorder as a conformational characteristic. There are other predictors that could be used to expand the approach in the future and new predictors are continuously being developed as more is learned about how protein functionality is coupled to the conformational flexibility of proteins. Examples are the s2D predictor [27], which predicts secondary structure elements in relation to random coil regions, and Dynamine [28], which predicts the rigidity of the peptide backbone throughout protein sequences, as well as the ANCHOR [29] and MoRFpred [30] predictors, which predict protein interaction sites. More recently developed predictors include prediction of protein regions involved in phase transitions [31], prediction of decomposed residue-by-residue solvation free energy [32] and prediction of residue-by-residue compactness/secondary structure [33]. Thus, it is easy to see that a battery of predictors could be used to reveal many different conformational aspects of protein sets encoded by groups of differentially regulated genes identified in transcriptome data. Databases like the Database of Disordered Protein Prediction (D^2^P^2^) [34] or the more recently developed MobiDB [35], which contain collections of prediction data from different sources, will be useful tools for this purpose.

## 4. Materials and Methods

### 4.1. Data

Human protein regions predicted to be disordered and related data were downloaded from the publically available D^2^P^2^ database (available online: http://d2p2.pro/search/build) on 11 September 2017. Default options were used for the download except that “Genome” was set to “Homo sapiens 63_37” and the “Limit to” option was set to “all”. The downloaded data contained all predicted IDRs detected in a total of 917,132 features for each of 9 different intrinsic disorder predictors (Espritz_Disprot, Espritz_NMR, Espritz_Xray, IUPred_long, IUPred_short, PV2, PrDOS, VL-XT, and VSL2b). See the D^2^P^2^ website (available online: http://d2p2.pro) or [34] for details. Mean fold-change transcriptome values for 1050 genes that show significantly altered transcript levels when Jeko-1 mantle lymphoma cells adhere to MS-5 stromal cells were taken from a recently published study from our group [6].

### 4.2. Data Analysis

Data were imported into and analysed using the *R* statistical programming platform (version 3.4.3, https://cran.r-project.org) [36] using packages shipped with the standard version, together with the following additional packages: data.table [37], nortest [38], formattable [39], org.Hs.eg.db [40].

To match gene expression data to IDR data for proteins in the D^2^P^2^ data set, it was first necessary to match an ENSEMBL protein id (from the EMSEMBL database, http://www.ensembl.org/index.html) to each of the genes identified in the RNAseq experiment. This was done by matching entries in the RNAseq data with entries in the org.Hs.eg.db annotation database from which fields for ENSEMBL protein id (ENSEMBLPROT) and gene name (SYMBOL) were extracted and appended to the RNAseq data using ENTREZID as a common key. 18,686 of 23,445 entries in the RNAseq data set were matched and also had identical gene names. This set was used in the further analysis. The annotation for the vast majority of the non-matched genes indicated that they represented non-protein-coding genes, putative protein encoding genes or pseudogenes. 1009 of the 1050 adhesion regulated genes were matched to an ENSEMBL protein id and a control set of 17,612 genes that were not shown to be regulated by adhesion were uniquely matched. Entries for which “SEQID” in the D^2^P^2^ IDR data matched the ENSEMBL protein id in sets or subsets of the adhesion-regulated genes or non-regulated genes were used for analysis of the sets or subsets. Only D^2^P^2^ entries for IDRs ≥ 30 amino acid residues were used and data for the 9 different IDR predictors were extracted from the database and analysed separately.

Differences in IDR number between test sets and control sets were evaluated statistically by *z*-scores and associated *p*-values calculated from the measured test value compared to the mean of 1000 control values, calculated from 1000 re-samples (with replacement) randomly selected from the control data. The size of the control re-samples was the same as the size of the test set. Differences in IDR length between test sets and control sets were evaluated statistically using a Mann–Whitney test, a non-parametric test appropriate for non-normally distributed data. *p*-values were adjusted for multiple testing using the false discovery rate method.

## Figures and Tables

**Table 1:** Intrinsically disordered regions are enriched in proteins encoded by down-regulated genes in lymphoma cells upon adherence to stromal cells.

Gene Set Comparison	Espritz-D ^#^	Espritz-N ^#^	Espritz-X ^#^	IUPred-L ^#^	IUPred-S ^#^	PrDOS ^#^	PV2 ^#^	VLXT ^#^	VSL2b ^#^
Adsu vs. Nadsu									
IDR number in adsu	487	1638	1210	1462	1517	2353	2445	2361	1966
IDR number in nadsu *	382	1036	796	847	892	1520	1794	1492	1431
Adjusted *p*-value	2.38 × 10^−8^	1.32 × 10^−27^	2.81 × 10^−23^	4.34 × 10^−27^	1.32 × 10^−27^	4.24 × 10^−34^	2.85 × 10^−19^	2.75 × 10^−25^	8.29 × 10^−19^
**Adsu_Down vs. Adsu_Up**									
IDR number in adsu_down	276	1072	760	983	1025	1507	1508	1572	1216
IDR number in adsu_up *	171	458	363	387	397	758	683	639	606
Adjusted *p*-value	3.51 × 10^−78^	<1.00 × 10^−99^	<1.00 × 10^−99^	<1.00 × 10^−99^	<1.00 × 10^−99^	<1.00 × 10^−99^	<1.00 × 10^−99^	<1.00 × 10^−99^	<1.00 × 10^−99^

* mean of 1000 resamples of *n* proteins encoded by genes in nadsu or adsu_up, where *n* = the number of genes in adsu or adsu_down, respectively. Abbreviations: adsu (adhesion-regulated genes); nadsu (non-adhesion-regulated genes); adsu_down (down-regulated adsu); adsu_up (up-regulated adsu). ^#^ Predictors of intrinsic disorder that appear in the D^2^P^2^ database.

**Table 2:** Intrinsically disordered regions tend to be longer in proteins encoded by down-regulated genes in lymphoma cells upon adherence to stromal cells.

Gene Set Comparison	Espritz-D	Espritz-N	Espritz-X	IUPred-L	IUPred-S	PrDOS	PV2	VLXT	VSL2b
Adsu vs. Nadsu									
Median (mean) IDR length (adsu)	64 (125)	57 (97)	66 (98)	54 (87)	52 (67)	61 (98)	61 (90)	48 (62)	74 (128)
Median (mean) IDR length (nadsu)	61 (98)	56 (91)	62 (93)	54 (86)	51 (68)	55 (86)	56 (82)	47 (61)	66 (107)
Adjusted *p*-value *	6.04 × 10^−2^	7.33 × 10^−2^	**2.50** **× 10^−2^**	6.02 × 10^−1^	5.10 × 10^−1^	**3.75** **× 10^−11^**	**4.97** **× 10^−9^**	**2.42** **× 10^−2^**	**4.97** **× 10^−9^**
**Adsu_Down vs. Adsu_Up**									
Median (mean) IDR length (adsu_down)	68.5 (154)	60 (104)	76 (108)	56 (93)	53 (70)	64 (107)	65 (96)	49 (65)	79.5 (142)
Median (mean) IDR length (adsu_up)	59 (89)	54 (86)	58 (83)	50 (75)	50 (62)	58 (85)	57 (80)	46 (57)	67 (104)
Adjusted *p*-value *	**1.24** **× 10^−3^**	**5.26** **× 10^−3^**	**2.14** **× 10^−7^**	**5.08** **× 10^−3^**	**1.66** **× 10^−2^**	**3.38** **× 10^−4^**	**1.42** **× 10^−5^**	**1.73** **× 10^−3^**	**7.05** **× 10^−5^**

* Mann–Whitney test; bold text = *p* < 0.05. Abbreviations: adsu (adhesion-regulated genes); nadsu (non-adhesion-regulated genes); adsu_down (down-regulated adsu); adsu_up (up-regulated adsu).

**Table 3:** Distribution of IDRs predicted by VSL2b in proteins encoded by genes that are differentially regulated in lymphoma cells upon interaction with stromal cells.

Gene Set Comparison	Number of Proteins	Number (%) of Completely Disordered Proteins	Number (%) of Proteins with IDR	Median Percent IDR Per Protein (All Proteins)	Median Percent IDR Per Protein (IDR-Containing Proteins)
adsu_down	445	19 (4.3)	367 (82.5)	38.1	48.1
adsu_up	556	11 (2)	370 (66.5)	18.1	37.6
nadsu	17,459	476 (2.7)	11,248 (64.4)	17.8	37.6

adsu_down (down-regulated adhesion-regulated genes); adsu_up (up-regulated adhesion-regulated genes); nadsu (non-adhesion-regulated genes).
